# Development and Characterization of PEDOT:PSS/Alginate Soft Microelectrodes for Application in Neuroprosthetics

**DOI:** 10.3389/fnins.2018.00648

**Published:** 2018-09-19

**Authors:** Laura Ferlauto, Antonio Nunzio D’Angelo, Paola Vagni, Marta Jole Ildelfonsa Airaghi Leccardi, Flavio Maurizio Mor, Estelle Annick Cuttaz, Marc Olivier Heuschkel, Luc Stoppini, Diego Ghezzi

**Affiliations:** ^1^Medtronic Chair in Neuroengineering, Center for Neuroprosthetics and Institute of Bioengineering, School of Engineering, École Polytechnique Fédérale de Lausanne, Geneva, Switzerland; ^2^Tissue Engineering Laboratory, HEPIA, University of Applied Sciences and Arts Western Switzerland (HES-SO), Geneva, Switzerland

**Keywords:** conductive hydrogel, conjugated polymers, electrode array, electrochemistry, neural recordings

## Abstract

Reducing the mechanical mismatch between the stiffness of a neural implant and the softness of the neural tissue is still an open challenge in neuroprosthetics. The emergence of conductive hydrogels in the last few years has considerably widened the spectrum of possibilities to tackle this issue. Nevertheless, despite the advancements in this field, further improvements in the fabrication of conductive hydrogel-based electrodes are still required. In this work, we report the fabrication of a conductive hydrogel-based microelectrode array for neural recording using a hybrid material composed of poly(3,4-ethylenedioxythiophene)-poly(styrenesulfonate), and alginate. The mechanical properties of the conductive hydrogel have been investigated using imaging techniques, while the electrode arrays have been electrochemically characterized at each fabrication step, and successfully validated both *in vitro* and *in vivo*. The presence of the conductive hydrogel, selectively electrodeposited onto the platinum microelectrodes, allowed achieving superior electrochemical characteristics, leading to a lower electrical noise during recordings. These findings represent an advancement in the design of soft conductive electrodes for neuroprosthetic applications.

## Introduction

In neuroprosthetics, the realization of a performing interface between an implant and the soft tissue is of primary importance for both the preservation of the neuronal environment, and the correct functioning of the device (Lacour et al., 2016). The main approaches taken into consideration for this purpose are essentially two: the modification of the electrode surface with conductive polymers (CPs) or carbon nanotubes (CNTs) (Abidian et al., 2010; Guex et al., 2015; Charkhkar et al., 2016) and the encapsulation of the device in a hydrogel matrix (Kim et al., 2010; Heo et al., 2016). CPs and CNTs are versatile carbon-based materials already exploited in the fabrication of several neuroprosthetic devices (Antognazza et al., 2015; Khodagholy et al., 2015; Feyen et al., 2016; Xiang et al., 2016; Ferlauto et al., 2018). In particular, their incorporation in the active sites of recording or stimulating neural devices is known to lower the impedance and the stiffness of metal electrodes, to allow both electronic and ionic charge transport, and to promote cell adhesion and proliferation (Abidian et al., 2010; Harris et al., 2013; Balint et al., 2014; Martin and Malliaras, 2016; Rivnay et al., 2016; Simon et al., 2016). Despite these attractive properties, these materials still suffer from a Young’s modulus far higher than the one of the tissue they are in contact with, potentially leading to important implications in terms of foreign body reaction and ultimately to the device failure (Salatino et al., 2017).

On the other hand, hydrogels are natural, or synthetic materials based on a three-dimensional network of polymer chains that can retain a large amount of water and are already used in a variety of biomedical applications, ranging from contact lenses to wound healing, and drug delivery (Buwalda et al., 2014; Caló and Khutoryanskiy, 2015; Ullah et al., 2015; Bryant and Vernerey, 2018). Their tunable mechanical properties, high water content, high porosity, and soft consistency mimic the ones of living tissues; this makes hydrogels extremely attractive for neural prostheses.

To combine the strengths of these two approaches, the exploitation of conductive hydrogels (CHs) as coatings for microelectrodes in neural implants has recently emerged as a promising strategy (Green et al., 2012, 2016; Goding et al., 2017; Staples et al., 2017; Spencer et al., 2017). A CH on the electrodes could in fact simultaneously guarantee appropriate electrochemical and mechanical properties for the interaction with the neural tissue.

To our knowledge, despite recent progress in this field, a selective micro-sized confinement of CHs, with a thorough electrochemical characterization at each fabrication step, followed by *in vitro* and *in vivo* validation, is still missing (Kleber et al., 2017; Staples et al., 2017). The aim of this work is therefore to provide a comprehensive characterization of a CH-based microelectrode array, consisting of a poly (3,4-ethylenedioxythiophene)-poly(styrenesulfonate; PEDOT:PSS) and alginate (A) coating (called conductive alginate, CA) selectively electrodeposited on platinum (Pt) microelectrodes to reduce the mismatch of the material properties at the electrode-tissue interface.

## Materials and Methods

### Electrode Arrays Used in the Study

Pt-based microelectrode arrays (MEAs) with various geometries (linear or grid), electrode diameters (30, 100, or 400 μm), and electrode coatings (PEDOT:PSS, PEDOT:PSS/A, PEDOT:PSS/CA, and Pt Black) have been fabricated and used in this study. For structural and electrochemical characterization, planar grid MEAs (g-MEAs) with 16 electrodes (4 × 4) of 400 μm in diameter and a center-to-center distance of 1 mm were used. Electrodes were bare Pt, or Pt-coated with PEDOT:PSS, PEDOT:PSS/A, and PEDOT:PSS/CA. For *in vitro* validation, two planar MEAs with eight electrodes of 30 μm in diameter were used: a bottom porous MEA (p-MEA) and a top strip MEA (s-MEA). Pt electrodes were coated with Pt black or with PEDOT:PSS/CA. For *in vivo* validation, penetrating linear MEAs (l-MEAs) with 16 electrodes of 100 μm in diameter and a center-to-center distance of 200 μm were used. Electrodes were bare Pt or Pt-coated with PEDOT:PSS/CA.

### Electrode Array Fabrication

Microelectrode arrays were fabricated on 4-inch silicon (Si) wafers (thickness 525 μm) with a titanium-tungsten alloy/aluminum release layer (TiW/Al, 200 nm/1 μm). A polyimide (PI) layer (HD MicroSystems PI2611, 10 μm) was spin-coated (1,400 rpm for 40 s) and then cured by a soft bake (5 min at 65°C and 5 min at 95°C) followed by a hard bake (1 h at 300°C with nitrogen from 190°C). A titanium/platinum (Ti/Pt, 5 nm/150 nm) adhesive/conductive layer was deposited by sputtering (Alliance Concept AC450). A positive photoresist (AZ1512, 2 μm) was deposited by spin-coating and soft baked at 110°C for 2 min before direct exposure (Heidelberg Instruments MLA150, 405 nm) and development. Electrode shaping and photoresist removal were performed by chlorine dry etching (Corial 210IL) followed by oxygen plasma (500 W for 30 s). MEAs were encapsulated by spin-coating an adhesion promoter (VM651, 1,000 rpm for 10 s + 3,000 rpm for 30 s), spin-coating and soft baking a first PI layer (PI2611, 10 μm) followed by a second layer (10 μm), and curing (soft and hard bake). Then, a Si hard mask (1 μm) was deposited by sputtering (Alliance Concept AC450) and the photolithography was repeated. Dry etching (Corial 210IL) of Si and eventually PI and photoresist (respectively, chlorine and oxygen chemistries) allowed the exposure of Pt pads and electrodes. A final Si dry etching removed the remaining hard mask. An extra etching step was performed on p-MEAs to fabricate a porous substrate (7.5 μm diameters holes with a pitch of 20 μm). The MEAs were cut by a laser cutter (Optec MM200-USP) and released by Al anodic dissolution for 15 h.

### PEDOT:PSS Coating

An aqueous solution of 0.1 wt% 3,4-ethylenedioxythiophene (EDOT 97%, 483028, Sigma) and 4 wt% poly(4-styrenesulfonic acid) solution (PSS, M_w_ ˜75,000, 561223, Sigma) in deionized water was mixed by ultrasonication for 5 min before being filtered with 0.2 μm PTFE filters (431229, Corning). The electropolymerization of PEDOT:PSS was obtained using a potentiostat (Compact Stat, Ivium). MEAs were immersed in the solution together with a silver/silver-chloride (Ag/AgCl) reference electrode and a Pt counter electrode. The potential was increased from 0.4 to 0.9 V in 5 steps of 0.1 V and 2 s in duration. Then, the potential was held at 0.9 V for 40 s. The protocol was repeated twice and MEAs were finally cured at 65°C for 3 h.

### Alginate and Conductive Alginate Coating

1% Alginate mother solution was prepared by dissolving 1 wt% of alginic acid from brown algae (A2033, Sigma) and 0.5 wt% of calcium carbonate (CaCO_3_ -99%, C5929, Sigma) in phosphate-buffered saline (PBS). The solution was then stirred and heated at 100°C for 1 h and all further alginate deposition solutions with different concentrations were obtained by its dilution (Cheng et al., 2011). First, an adhesion layer composed of 0.1 wt% EDOT and 4 wt% PSS in alginate deposition solution was prepared, ultrasonicated for 5 min, and electropolymerized (as in section “PEDOT:PSS coating”). Then, alginate was electropolymerized from the alginate deposition solution with a voltage increase from 0 to 2 V without any intermediate step and kept constant for 2 s. MEAs were then stored in a hardening solution consisting of 1 wt% of calcium chloride (CaCl_2_, C7902, Sigma) in PBS for at least 30 min. To obtain a CA, the electropolymerization of PEDOT:PSS was then repeated twice (as in section “PEDOT:PSS Coating”).

### Platinum Black Coating

Platinum electrodes were coated with platinum black using a platinum solution made of: 2 g H_2_PtCl_6_ ⋅ xH_2_O, 16 mg C_4_H_6_O_4_Pb ⋅ 3H_2_O, and 58 g of H_2_O (Sigma). A 700 mV signal at 300 Hz was applied via a 4,284A Precision LCR Meter (Keysight Technologies) until the electrode impedance reached a magnitude of 8 kΩ and a phase of -45°. About 10–15 s were required to achieve sufficient plating corresponding to a black platinum coating thickness of 300 to 400 nm.

### PEGDMA Coating

The 5 wt% of Poly(ethylene glycol) dimethacrylate (PEGDMA, M_n_ 20,000, 25406-5, Polysciences), 5 wt% of PEGDMA (M_n_ 550, 409510, Sigma), and 0.5 wt% of 2-hydroxy-4′-(2-hydroxyethoxy)-2-methylpropiophenone photoinitiator (IRGACURE 2959, 410896, Sigma) were mixed in PBS and ultrasonicated until complete dissolution. MEAs were dipped in this solution for 3 s and then exposed to UV light (365 nm, Thorlabs) for 15 min. Afterward, MEAs were stored inside the hardening solution (1 wt% of CaCl_2_ in PBS).

### Microscopic Characterization

The thickness of the PEDOT:PSS double layer was measured using a Bruker’s DektakXT Stylus Profiler. The topography and the elastic modulus surface map were taken using a Dimension Icon atomic force microscope (AFM, Bruker); measurements were performed in liquid (1 wt% of CaCl_2_ in PBS), with a ScanAsyst Fluid + probe (Bruker, nominal spring constant 0.7 N m^-1^). Each scan contains 512 lines of 512 data points across a 3 μm × 3 μm surface. Images were analyzed using Gwyddion software. For each map, the average stiffness value with its SD was obtained. The variance of the stiffness was calculated as the square of the standard deviation.

### Electrochemistry

Electrochemical characterizations were performed with a three-electrode (Ag/AgCl reference electrode, Pt counter electrode) potentiostat (Compact Stat, Ivium) in PBS (pH 7.4) at room temperature. Impedance spectroscopy (IS) was performed between 1 Hz and 1 MHz with an AC voltage of 50 mV. Cyclic voltammetry (CV) was obtained by sweeping a cyclic potential at a speed of 50 mV s^-1^ between -0.6 and 0.8 V for Pt electrodes and between -0.9 and 0.8 V for coated electrodes. For each electrode, the average response over 5 cycles was calculated; the anodic and cathodic charge storage capacities (CSCs) were extrapolated from the integration of the respective currents.

### Cell Culture

Human neural stem cells from induced pluripotent stem cells (iPSCs) were obtained from MTI-Globalstem (GSC-4301, Thermo Fisher Scientific). They were cultured and maintained on 1:200 GelTrex LDEV-free hESC quality (A1413302, Thermo Fisher Scientific) coated flask in a proliferation medium composed of neurobasal^TM^ medium (21103049, Thermo Fisher Scientific) supplemented with 2% B-27^TM^ supplement (17504001, Thermo Fisher Scientific), MEM non-essential amino acids (11140050, Thermo Fisher Scientific), 1% Glutamax^TM^ supplement (35050061, Thermo Fisher Scientific), and 20 ng ml^-1^ FGF-2 (100-18B, PeproTech). For the generation of three-dimensional neurospheres (NSs), cells were detached at approximately 80% confluence with pre-warmed StemPro^TM^ Accutase^TM^ (A1110501, Thermo Fisher Scientific) for 1–2 min. The single cell suspension was centrifuged for 3 min at 320 g, suspended in proliferation medium and cells were counted. 500,000 cells in 3 ml proliferating medium were added into a non-treated six-well plate. The plate was left under orbital agitation (80 rpm) for 4 days in a cell culture incubator at 37°C (100% humidity, 5% CO_2_). 24 h later, the free-floating three-dimensional NSs were formed by aggregation. Four days after seeding, the NS size was checked and switched to a differentiation medium composed of NeuralQ^TM^ Basal Medium (GSM9420, GlobalStem), GS21T Supplement (GSM3100, GlobalStem), and 1% Glutamax^TM^ supplement (35050061, Thermo Fisher Scientific). Cultures were maintained in orbital agitation (80 rpm) for 6 weeks. A breathable plate sealer was added in order to reduce medium evaporation. The medium was changed once a week (3 ml). Mature NSs were then transferred to a 6 mm patch of pre-cut circular hydrophilic membrane supported by a six well insert (PICMORG50, Merck-Millipore) and kept in the incubator for 1 week. The use of membrane pre-cut patches facilitates the NS manipulation.

### Electrophysiology *in vitro*

Neurospheres on membranes were transferred with forceps under a dissection microscope (Leica Microsystems) onto the center of a p-MEA device. After 1 day of recovery, NSs top surface was put in contact with an s-MEA device (coated with platinum black or conductive alginate) and electrophysiological recordings were performed using an amplifier (W2100-HS32, Multi Channel Systems) and a data acquisition system (W2100, Multi Channel Systems). The signal-to-noise ratio (SNR) was evaluated as follow: for each electrode, the noise was quantified as the standard deviation of the voltage during a 5 min recording, while the signal was the average peak-to-peak voltage of the spikes recorded in the same 5 min period. For each electrode, the SNR was computed as the signal divided by the noise. Therefore, electrodes unable to detect neural spikes have been excluded (*n* = 11 out of 16 and *n* = 4 out of 21, respectively, for s-MEA with Pt black and s-MEA-CA).

### Electrophysiology *in vivo*

Animal experiments were performed according to the animal authorization GE13416 issued by the Département de l’emploi, des affaires sociales et de la santé (DEAS), Direction générale de la santé of the Republique et Canton de Geneve, Switzerland. Two months old C57BL6J mice were anesthetized with isoflurane inhalation (induction 0.8–1.5 l min^-1^, 4–5%; maintenance 0.8–1.5 l min^-1^, 1–2%). A 0.5 mm small craniotomy was opened in the correspondence of the visual cortex (identified by stereotaxic coordinates). MEAs were implanted in the cortical layers using a micromanipulator (SM-15R, Narishige). Light flashes (4 ms, 30 cd s m^-2^) were delivered using a Ganzfeld stimulator (Biomedica Mangoni) positioned close to the contralateral eye and visually evoked cortical potentials (VEPs) were recorded and filtered (0.1–300 Hz).

### Statistical Analysis and Graphical Representation

Statistical analysis and graphical representation were performed on Prism (GraphPad Software Inc.). The normality test (D’Agostino & Pearson omnibus normality test) was performed in each dataset to justify the use of a parametric or non-parametric test. In each figure *p*-values were represented as: ^∗^*p* < 0.05, ^∗∗^*p* < 0.01, ^∗∗∗^*p* < 0.001, and ^∗∗∗∗^*p* < 0.0001. Data were always reported as mean ± SD or ± SEM; *n* identifies the number of electrodes.

## Results

**FIGURE 1:**
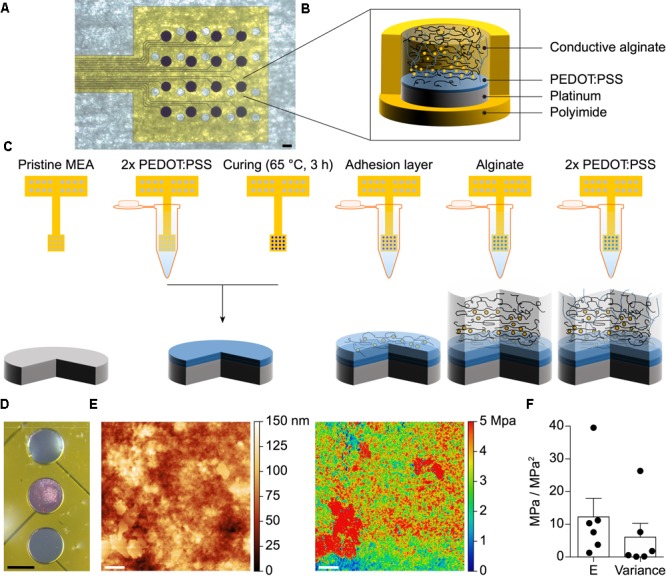
**(A)** Pictures of a fabricated g-MEA with 400 μm diameter electrodes. The scale bar is 400 μm. **(B)** Schematic of the soft CA-based electrode structure. **(C)** Step-by-step coating process of the CA-based electrodes. **(D)** Selective electroplating of alginate with added Rhodamine B on the central Pt electrode only. The scale bar is 150 μm. **(E)** AFM topography map of a 3 × 3 μm surface area of the CA (left) and the corresponding elastic modulus surface map (right). The scale bar is 400 nm. **(F)** Quantification of the mean (± SEM) stiffness and variance (*n* = 6 electrodes, 1 map per electrode).

Microelectrode arrays with various geometries (linear or grid) and electrode diameters (30, 100, or 400 μm) have been fabricated using PI as substrate and superstrate and Pt for the electrodes, traces, and pads (**Figure 1A**). Soft electrodes consisting of a first layer of PEDOT:PSS and a second layer of CA have been then electrodeposited on top of the Pt electrodes (**Figures 1B,C**). With a mechanical profilometer the average (± SD, *n* = 12) thickness of the PEDOT:PSS layer has been quantified as 1.18 ± 0.69 μm. The sharp confinement of the conductive alginate coating on top of the electrodes has been obtained by ionic crosslinking via electroplating (Cheng et al., 2011). This has been experimentally visualized and validated by adding Rhodamine B to the hydrogel (**Figure 1D**). The map of the elastic modulus on the surface of the CA (**Figure 1E**) has been obtained through peak-force quantitative nanomechanical mapping atomic force microscopy (PF-QNM AFM) of the hydrated sample. The variability of the modulus values points out the presence of an interpenetrating network of a stiffer material within a softer embedding, thus revealing the co-presence of the two components of the CA. The mean (± SEM) elastic modulus measured from various electrodes (*n* = 6) has been quantified in 12.29 ± 5.67 MPa with a mean (± SEM) variance of 6.10 ± 4.21 MPa^2^ (**Figure 1F**).

**FIGURE 2:**
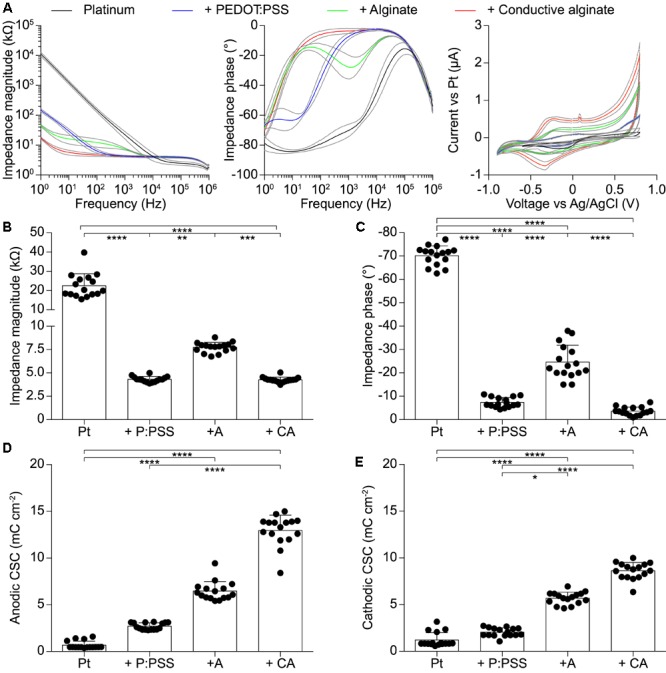
Quantification of the electrochemical characteristics of g-MEA with 400-μm diameter electrodes (*n* = 16) at each fabrication step. **(A)** IS (magnitude and phase) and CV performed at each fabrication step. Colored lines represent average values and gray lines the ± SD. **(B)** Impedance magnitude at 1 kHz (*p* < 0.0001, Kruskal–Wallis test with Dunn’s multiple comparisons test). **(C)** Impedance phase at 1 kHz (*p* < 0.0001, one-way ANOVA with Tukey’s multiple comparisons test). **(D)** Anodic CSC (*p* < 0.0001, Kruskal–Wallis test with Dunn’s multiple comparisons test). **(E)** Cathodic CSC (*p* < 0.0001, Kruskal–Wallis test with Dunn’s multiple comparisons test). In all panels P:PSS means PEDOT:PSS.

**FIGURE 3:**
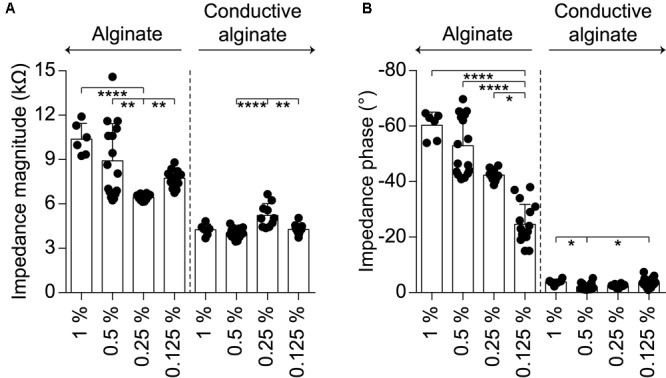
Quantification of the electrochemical characteristics of g-MEA with 400-μm diameter electrodes at different alginate concentrations (1%, *n* = 6; 0.5%, *n* = 16; 0.25%, *n* = 10; 0.125% *n* = 16); bare alginate on the left and CA on the right. **(A)** Impedance magnitude at 1 kHz (alginate *p* < 0.0001, CA *p* < 0.001, Kruskal–Wallis test with Dunn’s multiple comparisons test). **(B)** Impedance phase at 1 kHz (alginate *p* < 0.0001, CA *p* < 0.01, one-way ANOVA with Tukey’s multiple comparisons test).

IS and CV have been performed at each fabrication step on g-MEAs with 400 μm electrodes to evaluate their electrochemical characteristics (**Figure 2**). In agreement with the literature (Staples et al., 2017), CA-based electrodes showed (mean ± SD, *n* = 16) a lower impedance magnitude (4.26 ± 0.29 kΩ at 1 kHz), a resistive behavior (-3.59 ± 1.71° at 1 kHz), and a larger charge storage capacity (CSC, anodic: 12.96 ± 1.63 mC cm^-2^, cathodic: 8.62 ± 0.90 mC cm^-2^) in comparison with the other conditions tested (bare Pt, Pt-coated with PEDOT:PSS, and Pt-coated with PEDOT:PSS and pure alginate). Amongst the different concentrations of alginate within the conductive hydrogel, the lowest (0.125%) has been chosen due to its effectiveness in constraining the gel on the electrode sites. In fact, higher gel concentrations will form a continuous layer over the entire electrode array due to the higher viscosity of the solution. From the electrochemical point of view, an improvement of impedance magnitude and phase of bare alginate coated electrodes was observed by lowering the alginate concentration, while no effect was appreciable on CA-based electrodes (**Figure 3**).

The detailed characterization of the CA-based electrodes has been performed at 1 kHz since this frequency is a standard reference for application in neuroprosthetics. It should be noted (**Figure 2A**), that at lower frequencies (<100 Hz), the mean impedance magnitude of PEDOT:PSS coated electrodes starts to increase, while the mean impedance magnitude of CA-based electrodes remains remarkably low till a frequency of 10 Hz. Below 10 Hz, it starts to increase but remains at values which are 10 times lower than those of PEDOT:PSS coated electrodes. This could be very relevant for several biomedical applications in which low-frequency signals are collected, such as for electrocardiographic and electromyographic tattoo skin sensors (Zucca et al., 2015; Wang et al., 2018).

**FIGURE 4:**
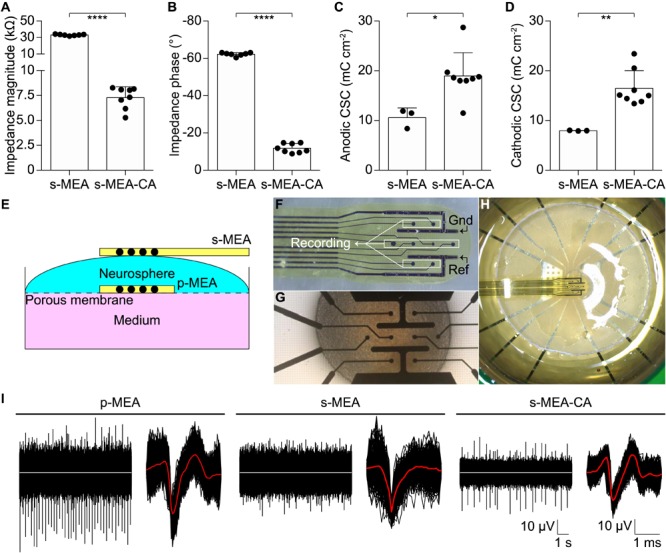
Validation *in vitro*. **(A–D)** Quantification of the impedance magnitude at 1 kHz (*p* < 0.0001, *t*-test), impedance phase at 1 kHz (*p* < 0.0001, *t*-test), anodic CSC (*p* < 0.05, *t*-test), cathodic CSC (*p* < 0.01, *t*-test) of s-MEA (*n* = 7 for IS and *n* = 3 for CV) and s-MEA-CA (*n* = 8). The electrode diameter is 30 μm. **(E)** Sketch of the recording set-up. **(F)** Picture of an s-MEA. **(G)** Picture of a p-MEA with the NS on top. **(H)** Picture of the set-up with p-MEA, NS, and s-MEA. **(I)** Representative recordings from a p-MEA, an s-MEA, and an s-MEA-CA. For each, on the left is a representative raw trace and on the right the overlay of the spike waveform from the same electrode. The white traces represent the baseline (0 μV), while the red lines are the averages of the spike waveforms.

**FIGURE 5:**
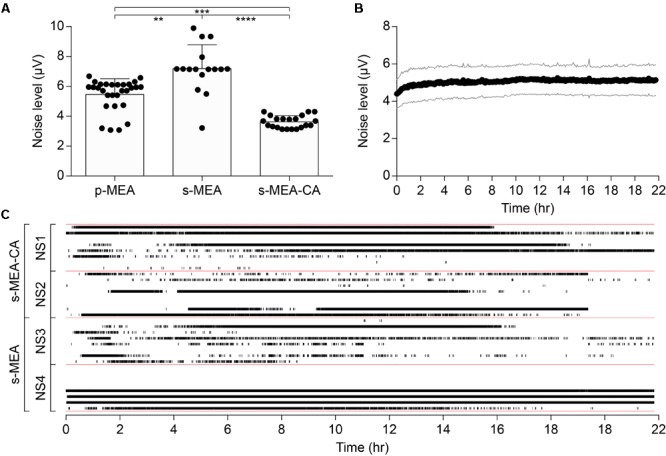
Noise performance of CA-based electrodes. **(A)** Mean (± SD) of the noise level between p-MEA (*n* = 29), s-MEA (*n* = 16), and s-MEA-CA (*n* = 21); *p* < 0.0001 Kruskal–Wallis test with Dunn’s multiple comparisons test. **(B)** Mean (± SD) noise level over the time of s-MEA-CA (*n* = 21). **(C)** Spike raster plot of four NSs over 22 h of recordings. s-MEA (two NSs) and s-MEA-CA (two NSs) have eight electrodes each.

The performance of the CA-based electrode array in recording neuronal signals has been tested *in vitro* on NSs formed by human neural stem cells derived from iPSCs. CA-based electrodes (30 μm in diameter) have been compared to Pt/Pt black ones, which represent the standard reference in neuronal recordings. As in the previous case, the electrochemical characterization of CA-based electrodes showed (mean ± SD, *n* = 8) a lower impedance magnitude (7.29 ± 1.09 kΩ at 1 kHz), a more resistive behavior (-11.82 ± 2.46° at 1 kHz), and an increase in the CSC values (anodic: 18.96 ± 4.68 mC cm^-2^, cathodic: 16.46 ± 3.57 mC cm^-2^) with respect to Pt/Pt black electrodes (**Figures 4A–D**). For *in vitro* validation, four NSs have been placed onto four p-MEAs with 30 μm Pt/Pt black electrodes, used as a reference for tissue viability. The top side of each NS has been contacted with an s-MEA embedding either 30 μm Pt/Pt black (s-MEA) or 30 μm CA-based (s-MEA-CA) electrodes (**Figures 4E–H**). Both p-MEA and s-MEA showed a noise level larger than s-MEA-CA (**Figure 4I**), that has been quantified as the standard deviation of the voltage recordings. In agreement with IS (**Figure 4A**), CA-based electrodes (s-MEA-CA) showed a better noise level (**Figure 5A**) compared to Pt/Pt black electrodes (p-MEA and s-MEA). Since NSs are seeded on top of the p-MEA, they had a stronger adhesion to the tissue, which translated into the detection of spikes with a higher peak-to-peak voltage with respect to any s-MEA (with or without CA). On the contrary, the s-MEA suffered from a weak contact to the NSs due to their spherical surface and the flexibility of the PI strip; this resulted in the detection of spikes with a lower peak-to-peak voltage (**Figure 4I**). However, regardless of the weaker contact, the s-MEA-CA devices presented a lower noise level in comparison with the s-MEA and p-MEA (**Figure 5A**), which turned into a better SNR with respect to the equivalent condition with s-MEAs: mean SNRs (± SD) are 6.75 ± 0.87 (*n* = 5) and 8.20 ± 1.35 (*n* = 17), respectively, for s-MEA with Pt black and s-MEA-CA (*p* < 0.05, unpaired *t*-test). Moreover, the noise level of s-MEA-CA devices was not affected over a time-period of 22 h (**Figure 5B**), in which neuronal spikes have been detected (**Figure 5C**). The frequency of the detected spikes decreases after about 16 h of recordings. This effect has not been associated with a deterioration of the CA-based electrodes since both electrode types (s-MEA and s-MEA-CA) showed a similar reduction. On the contrary, it could be owed to a reduction of the NS viability.

**FIGURE 6:**
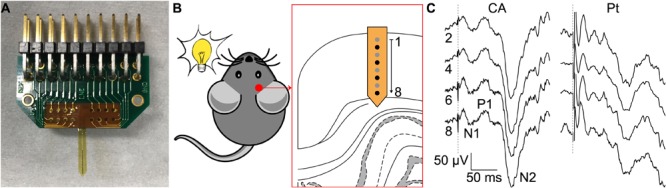
Validation *in vivo*. **(A)** Picture of a penetrating MEA with CA electrodes. **(B)** Experiment sketch. **(C)** Representative single-sweep recordings from 4 (out of 8) CA-based electrodes (diameter 100 μm, black electrodes in **B** from an implanted array on the left and representative averaged recordings (average of 10 consecutive sweeps) from 4 (out of 8) bare Pt electrodes (diameter 100 μm) on the right. The dotted gray line represents the flash occurrence (4 ms, 30 cd s m^-2^). The main peaks of the VEP are highlighted in the trace of electrode 8.

**FIGURE 7:**
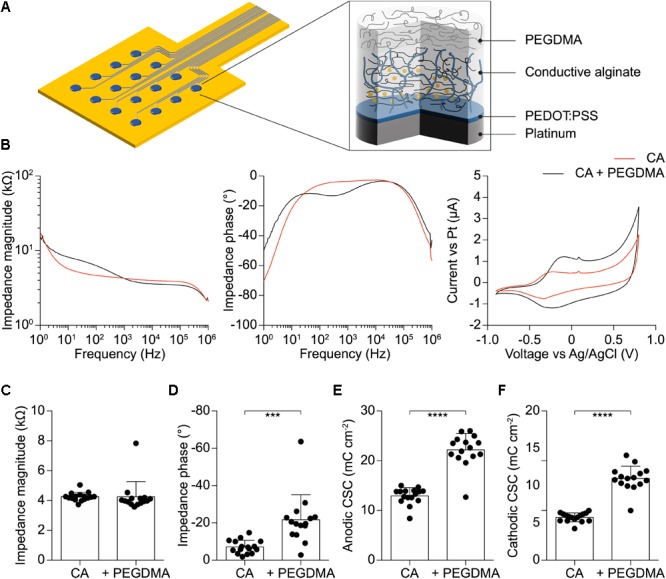
**(A)** Schematic of the soft electrode structure coated with PEGDMA. **(B)** Mean impedance magnitude, phase, and cyclic voltammetry for CA (red) and PEGDMA (black) 400 μm diameter electrodes (*n* = 15). **(C)** Quantification of the impedance magnitude at 1 kHz (*p* = 0.9568, *t*-test, *n* = 15). **(D)** Quantification of the impedance phase at 1 kHz (*p* < 0.001, *t*-test, *n* = 15). **(E)** Quantification of the anodic CSC (*p* < 0.0001, *t*-test, *n* = 15). **(F)** Quantification of the cathodic CSC (*p* < 0.0001, *t*-test, *n* = 15).

The ability of CA-based electrodes to detect neuronal activity has also been proven *in vivo*. Penetrating CA-based MEAs with linear geometry and electrode diameters of 100 μm have been implanted in the visual cortex of mice (**Figure 6A**). Due to the thin thickness of the mouse visual cortex, only the first eight electrodes of the probe (16 in total) have been inserted over the entire cortical thickness (**Figure 6B**). VEPs have been successfully recorded upon light stimulation of the contralateral eye (**Figure 6C**, left). Because of the low impedance of the CA electrodes, individual responses to a single flash highlighted all main components (positive and negative peaks, respectively, P and N) of the cortical VEP, without synchronous averaging. As a qualitative comparison, the cortical VEP recorded with bare Pt electrodes (diameter of 100 μm) upon synchronous averaging of 10 consecutive responses have been shown (**Figure 6C**, right). Qualitatively, it is visible that CA-based electrodes have better performances even without synchronous averaging. Lastly, taking into consideration the possibility of the alginate to dissolve with time during chronic implantation (Lee and Mooney, 2012), we also verified that a protective coating of the probe with Poly(ethylene glycol) dimethacrylate (PEGDMA), created by dip-coating, would not alter the electrochemical properties of the CA-based electrodes (**Figure 7**).

## Conclusion

The use of CA as a soft coating of MEAs is an attractive strategy to both reduce the mechanical mismatch at the electrode-tissue interface and improve the electrochemical properties of microelectrodes (Green et al., 2012, 2016; Goding et al., 2017). Our results showed a selective and sharp micro-sized confinement of CA onto the metallic electrodes of MEAs, which is one of the open challenges in the field. In addition, we provided a comprehensive and complete characterization of CA-based MEAs via electrochemical, mechanical, and electrophysiological analyses. With respect to Pt and Pt/Pt black electrodes, which are the standard materials employed in clinical devices, the soft CA-based microelectrodes presented in this work demonstrated lower impedance magnitude, higher CSCs, and a more resistive behavior. This turns into an improved SNR during neuronal recordings. These results represent an important advancement for the fabrication of performing neuroprosthetic devices able to reduce the mechanical and electrical mismatch at the electrode-tissue interface.

## Data Availability Statement

The authors declare that all the data supporting the findings of the study are available in this article. Access to our raw data can be obtained from the corresponding author upon reasonable request.

## Author Contributions

LF designed, fabricated, and tested the MEAs, co-supervised the study, and wrote the manuscript. AD optimized the conductive hydrogel coating and performed the electrochemical characterizations. PV performed the *in vivo* electrophysiology. MA fabricated the MEAs and optimized the electrodeposition of the PEDOT:PSS. FM performed and analyzed the *in vitro* electrophysiology. EC contributed to the electrochemical characterization. MH fabricated the Pt-black MEAs. LS designed the *in vitro* assay. DG designed and led the study, validate the data analysis, and wrote the manuscript. All the authors read and accepted the manuscript.

## Conflict of Interest Statement

The authors declare that the research was conducted in the absence of any commercial or financial relationships that could be construed as a potential conflict of interest.
